# Clustering of Genetic Anomalies of Cilia Outer Dynein Arm and Central Apparatus in Patients with Transposition of the Great Arteries

**DOI:** 10.3390/genes13091662

**Published:** 2022-09-16

**Authors:** Marlon De Ita, Javier Gaytán-Cervantes, Bulmaro Cisneros, María Antonieta Araujo, Juan Carlos Huicochea-Montiel, Alan Cárdenas-Conejo, Charles César Lazo-Cárdenas, César Iván Ramírez-Portillo, Carina Feria-Kaiser, Leoncio Peregrino-Bejarano, Lucelli Yáñez-Gutiérrez, Carolina González-Torres, Haydeé Rosas-Vargas

**Affiliations:** 1 Unidad de Investigación Médica en Genética Humana, UMAE Hospital de Pediatría, Centro Médico Nacional Siglo XXI, IMSS, Ciudad de México 06720, Mexico; 2 Dpto de Genética y Biología Molecular, CINVESTAV Zacatenco IPN, Ciudad de México 07360, Mexico; 3 Laboratorio de Secuenciación, División de Desarrollo de la Investigación, IMSS, Ciudad de México 06720, Mexico; 4 Departamento clínico de Genética Médica, UMAE Hospital de Pediatría, Centro Médico Nacional Siglo XXI, IMSS, Ciudad de México 06720, Mexico; 5 Departamento clínico de Cardiología, UMAE Hospital de Pediatría, Centro Médico Nacional Siglo XXI, IMSS, Ciudad de México 06720, Mexico; 6 Unidad de Cuidados Intensivos Neonatales, UMAE Hospital de Pediatría, Centro Médico Nacional Siglo XXI, IMSS, Ciudad de México 06720, Mexico; 7 UMAE Hospital de Pediatría, Centro Médico Nacional Siglo XXI, IMSS, Ciudad de México 06720, Mexico; 8 Clínica de Cardiopatías Congénitas, UMAE Hospital de Cardiología, CMN Siglo XXI, Ciudad de México 06720, Mexico

**Keywords:** cilia, whole-exome sequencing, transposition of great arteries, dynein, genetic counseling

## Abstract

Transposition of the great arteries (TGA) is a congenital heart defect with a complex pathogenesis that has not been fully elucidated. In this study, we performed whole-exome sequencing (WES) in isolated TGA-diagnosed patients and analyzed genes of motile and non-motile cilia ciliogenesis and ciliary trafficking, as well as genes previously associated with this heart malformation. Deleterious missense and splicing variants of genes *DNAH9*, *DNAH11*, and *ODAD4* of cilia outer dynein arm and central apparatus, *HYDIN*, were found in our TGA patients. Remarkable, there is a clustering of deleterious genetic variants in cilia genes, suggesting it could be an oligogenic disease. Our data evidence the genetic diversity and etiological complexity of TGA and point out that population allele determination and genetic aggregation studies are required to improve genetic counseling.

## 1. Introduction

Transposition of the great arteries (TGA) is a severe congenital heart disease (CHD) characterized by concordant atrioventricular and ventriculoarterial discordant connections in situs solitus. Although several monogenic syndromes have been associated with this defect, including Kabuki, Carpenter, and MRFACD, the etiology in its isolated and sporadic form is still a matter of debate [1,2,3]. Knockout mouse models of genes involved in left-right (LR) establishment have helped link TGA pathogenesis to an abnormal NODAL/GDF1-DAND5 developmental pathway (NGD).

NGD signaling depends on fluid flow gradients, with beating cilia being relevant to establishing LR asymmetry. Besides this, cilia have been associated with laterality establishment and heart development in other ways: the bending of primary cilia by hydrodynamic forces, the position of ciliary cells in the organizer, and the ciliary cell polarization in the posterior region of the embryo for LR establishment [4]. This evidence suggests that cilia function could be relevant in TGA pathogenesis.

In this regard, several cilia genes have been associated with this abnormal LR establishment and CHD. Abnormalities in the OFD1 gene, which is involved in ciliogenesis and the function of motile and non-motile cilia, have been associated with abnormal node development and the establishment of LR [5]. In motile cilia, alterations in heavy chain dyneins such as DNAH9 and DNAH11 have been associated with abnormal cilia beating and linked to congenitally corrected TGA [6,7,8]. Similarly, abnormalities in the intermediate strand DNAI1, required for cilia motility, have also been reported in patients with TGA [9,10]. In addition to these functions, primary cilia modulate several signaling pathways involved in cardiogenesis, such as TGF β and Sonic Hedgehog, whose alterations are related to outflow tract defects [11,12,13].

In general, congenital heart diseases, including TGA, have been linked with ciliopathies, such as Ellis van Creveld and Simpson–Golabi–Behmel syndrome [5,6,7]. However, a massive WES analysis recently suggested that cilia genetic anomalies could be relevant to TGA etiology [8]. Furthermore, the interaction and clustering of several deleterious genetic variants could lead to a predisposition with the transposition of the great arteries, as has been previously suggested for other CHD, such as the tetralogy of Fallot [9]. This study shows a high throughput analysis of genes previously linked with TGA (NODAL-GDF1-DAND5 signaling pathway) and motile (inner, outer dynein arms, and central apparatus) and non-motile cilia genes, which providence evidence for the identification of the etiology underlying this congenital heart disease.

## 2. Materials and Methods

### 2.1. Patients

Eleven patients, from newborn to 16 years, with transposition of the great arteries, were recruited from Pediatric and Cardiology Hospitals from National Medical Center XXI century, Mexican Social Security Institute (IMSS), Mexico. Clinical data relevant to this study were obtained from medical records. Given the presence of clinical manifestations suggestive of congenital heart disease, the diagnosis of transposition of the great arteries was made by pediatric cardiologists and described by ICD categorization. The patients were also evaluated by clinical geneticists to rule out the presence of other phenotypic alterations that would make it possible to integrate the diagnosis of various syndromic entities that may involve this type of congenital heart disease. Patients with congenital anomalies suggestive of syndromic entities were excluded from the study. The Research Ethics Board of the Mexican Social Security Institute, México, approved this study (R-2018-785-047). Prior to conducting this study, the parents of the affected children were informed and asked to give their consent via a signed written consent before the beginning of the study. Children eight years old and older signed an assent/consent document.

### 2.2. Sample Collection and Processing DNA Extraction

Blood samples were collected from 11 subjects diagnosed with TGA for WES. According to the manufacturer’s instructions, the genomic DNA was extracted using a QIAamp DNA Blood Mini Kit I (Qiagen, Hilden, Germany). DNA concentration and purity were measured using a Nanodrop Spectrophotometer (Thermo Fisher Scientific, Waltham, MA, USA) and Qubit 2.0 Fluorometer (Thermo Fisher Scientific, Waltham, MA, USA), while integrity was established by capillary electrophoresis using the TapeStation4200 (Agilent Technologies, Santa Clara, CA, USA).

### 2.3. Whole-Exome Sequencing

A total of 50 ng of genomic DNA per sample was used for DNA library preparation. DNA libraries were prepared by fragmentation using a Nextera DNA Exome sample prep kit (Illumina Inc., San Diego, CA, USA; Cat N. 20020616), involving end repair, A-tailing, and adapter ligation, as instructed by the manufacturer. Libraries were quantified using Qubit (dsDNA HS assays, ThemoFisher Scientific, Waltham, MA, USA, Cat.N. Q33230), pooled in equimolar amounts, and subjected to exome capture using XGen Research Panel version 1.0 (Integrated DNA Technologies, IDT, Coralville, IA, USA), with blocking adapters (xGen Blockers, IDT, Coralville, IA, USA), designed to match the Nextera library constructs following the manufacturer’s instructions. The libraries were diluted for sequencing on an Illumina Nextseq 500 platform using 2 × 146 paired-end cycles. BCL2 data were transformed to fastq through Bcl2fastq software, for further analysis, and the read quality of fastq files per sample was carried out using FAST-QC v0.11.9 under default parameters.

### 2.4. Variant Analysis

After WES, a DRAGEN Germline Pipeline (v3.2.8, Illumina, Inc., Waltham, MA, USA) was used to align the remaining high-quality sequencing reads to the reference human genome (hg19), and Germline Variant Small Hard Filtering (Illumina, Inc. Waltham, MA, USA) was used to annotate the identified single-nucleotide variants and InDels. The variant interpretation was performed with the Variant Interpreter software (Illumina Inc., Waltham, MA, USA). In the previously related genes, all non-synonymous variants were studied, whereas only VUS, pathogenic, or possibly pathogenic cilia genes were analyzed.

Nonsynonymous variants were then evaluated, determining their deleteriousness by *SIFT*, *PolyPhen2*, and a *Mutation Assessor* medium or high score. For splicing, nonsense, and indel variants, a LoF-Tool or CADD PHRED score was determined; a ≥20 value indicates that a variant is among the top 1% of damaging variants in the genome. Variant Effect Predictor was used to identify the effect on the canonic transcript.

To predict interactions, the STRING database was used (V11.5). First, a Markov Clustering Algorithm was employed to determine the global gene aggregation. Secondly, an interaction enrichment analysis was performed (EPv), where a *p*-value less than 0.05 was interpreted as indicative of a functional connection between proteins [10]. Protein domains were determined using Uniprot. Variants were subsetted to the *GnomAD* V2.1.1 to describe the Latin allele population frequency. As genetic variants presence interpretation is challenging, deleteriousness was defined using several algorithms. In silico analysis can predict whether a genetic anomaly will result in protein abnormalities that could lead to a malfunction or even its absence. The presence of deleterious variants may elevate the individual’s susceptibility or predisposition to the disease. Concerning missense variants, deleteriousness was determined by the presence of two abnormalities by *SIFT*, *PolyPhen2,* and *Mutation Assessor*, or the presence of one abnormality if a new variant was described. Although WES analysis is focused on exons, we analyzed proximal splicing variants when possible. Online Mendelian Inheritance in Man, *OMIM*^®^ McKusick-Nathans Institute of Genetic Medicine, Johns Hopkins University (Baltimore, MD, USA), (06-01-2022) was used to determine gene relevance in a clinical context. URL: https://omim.org/ (accessed on 30 November 2021). A list of the analyzed genes and their general relationship with motile and non-motile cilia, ciliogenesis, and ciliary trafficking can be found in Table S1.

### 2.5. Statistical Analysis

Probabilistic binomial analysis was performed to determine the probability of two or more variants in our cohort using the Minitab statistical software (Minitab19, Minitab LLC, State College, PA, USA). The variant expected population probability used in this analysis was obtained from frequency data from *GnomAD*. To compare the allele frequency between sexes, Exact Fisher Test was employed.

## 3. Results

### 3.1. Genetic Analysis Related to Syndromic Entities

**Figure 1:**
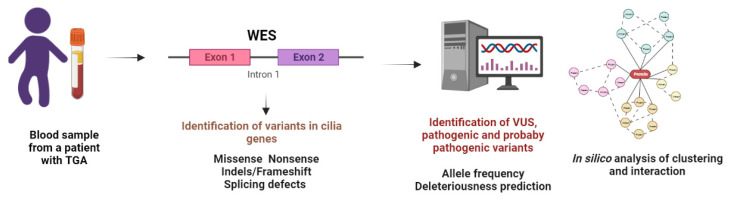
Genetic analysis abnormalities flow chart in patients with transposition of the great arteries. WES: Whole exome sequencing. VUS: Variants of Uncertain Significance.

We examined 11 unrelated probands with sporadic TGA, levocardia, and *situs solitus*. After WES analysis, a median of 160k variants per patient was obtained, and missense, nonsense, indels, or altered splicing regions were identified. A flow chart of the genetic analysis performed is presented in Figure 1.

First, genes previously associated with TGA in syndromic entities were analyzed (Table 1: Abnormalities in genes previously related to TGA)**.** Genetic anomalies associated with Kabuki syndrome were found in three probands: the first one carries a *KDM6A* hemizygous variant c.232C>T (p.R78C) OMIM Gene (OMIM-G) 300128, which was classified as non-deleterious (Table S2: Complete gene abnormalities of ciliary genes in TGA). The two remaining probands carry the autosomic *KMT2D* (OMIM-G 602113) deleterious variants c.547C>T (p.P183S) and c.15686G>A (p.R5229H). The first variant is in PHD finger 1 (PHD1), a module involved in the epigenetic regulation of gene expression via the recruitment of chromatin regulators and transcription factors [11]. The second variant is related to the FYR-C terminal domain of *KMT2D*, characterized by phenylalanine/tyrosine-rich regions found in various chromatin-associated proteins. The p.P183S is a newly described variant, while *KMT2D* p.R5229H is infrequently found in Latin American populations. It is worth noting that none of the patients displayed phenotypical characteristics of Kabuki syndrome.

On the other hand, variants of *MEGF8* (OMIM-G 604267), a gene related to TGA in Carpenter syndrome, were found in two patients. The first one carries the p.R439W (c.1315C>T) deleterious variant, while the second one carries the p.R782W non-deleterious variant, which is rare in the Latin American population (Table 1). However, both patients have heterozygous anomalies, whereas this entity is a recessive inheritance disorder.

### 3.2. NGD and Previously TGA-Related Genes

Next, TGA syndromic genes were analyzed, namely those related to LR establishment CHD (*NODAL*, *GDF1*, and *ACVR2B*) (Table 1). Several algorithms found two missense variants of the FOXH1 and GDF1 genes, which were classified as non-deleterious. Concerning ciliary genes previously associated with TGA, deleterious *DNAI1* and *DISC1* in T181101 and *SLC4A1* and *CLASP1* in T181201 variants were identified (Table 1). Since both patients were heterozygous carriers, we presume that different heterozygous deleterious anomalies in ciliary genes could have a summative effect, supporting an oligogenic etiology.

### 3.3. Motile and Non-Motile Genes, Ciliogenesis, and Cilia Trafficking Gene Analysis

To ascertain whether ciliary genes could be related to this CHD, we examined genes previously implicated with either functional or structural functions of motile cilia, non-motile cilia, and ciliary trafficking [12,13]. Several variants, including missense, nonsense codon, frameshift, and splicing alterations, were identified in *DNAH9*, *OFD1*, *BBS7*, and other genes (Table 2, Table 3, Table 4 and Table S2). Most ciliary deleterious genetic variants were found in heterozygous probands, except for homozygous probands for *HYDIN* and *PIBF1* and a hemizygous proband for *OFD1*. Only deleterious and possibly pathogenic variants were analyzed.

The most common anomalies were within the profile of the motile cilia (Table 2: Anomalies in motile ciliary genes in TGA patients), where patients displayed abnormalities in the outer dynein arm and central pair proteins. Deleterious variants of DNAH9 and DNAH11 genes were found in outer dynein arms genes. Four *DNAH9* variants were identified in three patients: a p.Y1017C and a p.G2384R variant; a splice donor splicing variant c.5151+1G>A and two missense changes in c.3050A>G. Although two patients displayed the same variant, the presence of 2 out of 22 alleles c.3050A>G in our sample has a probability of 0.2184, according to binomial probability calculation. Interestingly, the T180401 proband carries both missense and splicing variants that could have a summative effect in these patients. The latter variant was described as deleterious; as a result, the patient was compound heterozygous for the *DNAH9* gene. On the other hand, T180201 presented a DNAH11 nonsense heterozygous variant located in the C terminal domain, after the AAA6 region, in an area likely associated with a deleterious effect. As the allele presents a frequency of 2.897 × 10^−5^ in the Latin American population, a relationship with TGA is plausible in this case.

Additional deleterious genetic variants of outer dynein arms genes were found (Table 2). Two patients were hemizygous with deleterious variants in *OFD1*, a gene associated with Oral-Facial-Digital syndrome type 1 (OMIM Phenotype 311200), which has a sex-linked inheritance. A female proband carried an *OFD1* c.2610G>C (p.Q870H) variant, and a male proband had a c.1819C>T variant (p.F828V). Although p.Q870H has been observed in a male subject, the variant does not present a population difference between the sexes (exact Fisher test, *p* = 0.520), suggesting a lesser effect on a lethal cardiovascular phenotype. On the other side, the *OFD1* p.Q870H is a newly reported deleterious anomaly; however, it has been found in a female, so the effect of its probable deleteriousness could be masked. Finally, the presence of a novel homozygous *HYDIN* c.3332C>T (p.P1111L) deleterious variant in a T180401 proband was revealed by algorithm analysis.

Regarding non-motile cilia genes (Table 3: Genetic anomalies in non-motile cilia genes in TGA), a subject presented several abnormalities in genes related to this organelle as *INPPP5E* and *IQCE*, which are related to the modulation of Hedgehog signaling that could be relevant to outflow tract development [14,15,16]. On the other hand, a previously described pathogenic variant of the *PIBF1* gene was identified in three patients; two homozygous probands and a single heterozygous proband for c.1214G>A (p.R405Q) (Table 3). This gene has been associated with Joubert syndrome, a ciliopathy not associated with abnormal organ disposition (OMIM-P 617767). It is worth mentioning that finding 5 out of 22 alleles with a *p* < 0.05 according to binomial probability calculation with data of GnomAD for the Latin American population suggests that this is not a stochastic finding and could be associated with this disease.

Concerning ciliogenesis and cilia trafficking gene analysis (Table 4, genetic anomalies in ciliogenesis and ciliary trafficking in TGA patients), the patients presented several anomalies related to these processes. In addition to the presence of previously described *OFD1* and *PIBF1* genetic variants, *IFT46* anomalies were also observed. This gene is part of the IFT subcomplex B that is required for retrograde transport in the cilia but is also related to ciliogenesis [17,18]. In particular, *Ift46* KO mouse embryos displayed randomization of the embryo heart looping, a hallmark of defective lateralization; this effect has been associated with a lack of cilia in node cells [19]. The absence of nodal flow, which can be caused by the loss of nodal cilia and abnormal LR patterning, as seen in Ift46 embryos, has been consistently related to TGA [3].

### 3.4. Interaction and Clustering Analysis

**Figure 2:**
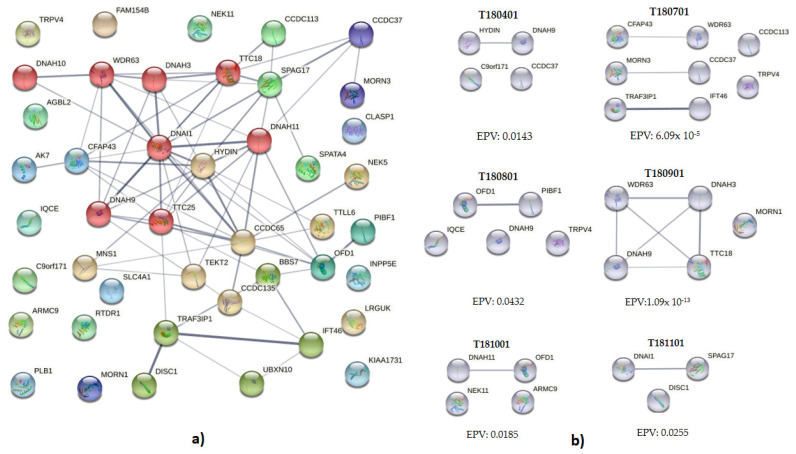
Genetic abnormality aggregation in patients with transposition of the great arteries. Global interaction of anomalies found in patients (**a**) The shared colors represent the clustering according to the Markov algorithm. Ciliary genetic anomalies are observed in genes involved in diverse processes such as ciliogenesis, ciliary trafficking, and the function of motile cilia. Individual patient aggregation of missense, nonsense, frameshift, and splicing deleterious variants is observed (**b**). In both, the line thickness indicates the strength of data support and the confidence of interactions; the thinnest line represents low confidence (0.150), next, medium confidence (0.400); then, high confidence (0.700), and, finally, the thickest line is the highest value (0.900). EPV. Enrichment *p*-value.

Apart from *DNAH9* and *HYDIN* variants found in some patients, most of the genetic variants were observed in genes related to autosomic recessive diseases. Thus, we hypothesized that clustering and interaction analyses might uncover an abnormal cilia function genotype by presenting several risk variants (Figure 2, genetic abnormality aggregation in patients with transposition of the great arteries). The interactions described by STRING software include direct (physical) and indirect (functional) associations, using knowledge transfer between organisms, and cluster interactions from other databases [10]. An interactive protein network was obtained in 29 out of 44 genes (65.9%) with deleterious variants (Figure 2a). Markov Clustering Algorithm displayed the presence of 7 different clusters, suggesting that dynamic protein complexes favor aggregation in static gene interactions (Figure 2a and Table S3). Further, to determine the cluster relevance, STRING interaction enrichment analysis was performed (EPV); a *p*-value of less than 0.05 was interpreted as indicative of a functional connection between proteins [10]. Using the EPV comprehensive analysis, a clustering includes deleterious variants of BBS7, TRAF3IP1, OFD1, and PIBF1 as part of primary cilia development (WP4536, STRING False Discovery Rate: 3.57 × 10^−8^). Interestingly, a cluster formed by 12 elements, including WDR63, DNAH3, DNAI1, TTC18, SPAG17, TTC25, and CCDC113, was found to be a cluster related to primary ciliary dyskinesia and COPI-independent Golgi-to-ER (Cluster 10689, STRING False Discovery Rate: 1.0–12) (Figure S1).

We next examined deleterious variants of TGA genes using an individual in silico functional interaction analysis. In 6 out of 11 analyzed probands (54.5%), clustering and interaction of anomalies were observed (Figure 2b). Abnormalities of DNAH9 and HYDIN interact in proband T180401, suggesting an oligogenic context in this patient. To T180701, several pairs of clustering anomalies, including CFAP43 and WDR63; CCDC36 and MORN3; TRAF3IP1 and IFT46. Studies using the single-celled model *Chlamydomonas* showed that the complex CFAP43/CFAP44 could regulate IDAF/I1(IC140), an orthologue of WDR63, hence, modulating the cilia beating [20,21]. Despite this, abnormalities of these genes have been implicated in nervous system malformations and infertility but not in heart defects [22,23,24,25], suggesting a poor association with TGA.

**Figure 3:**
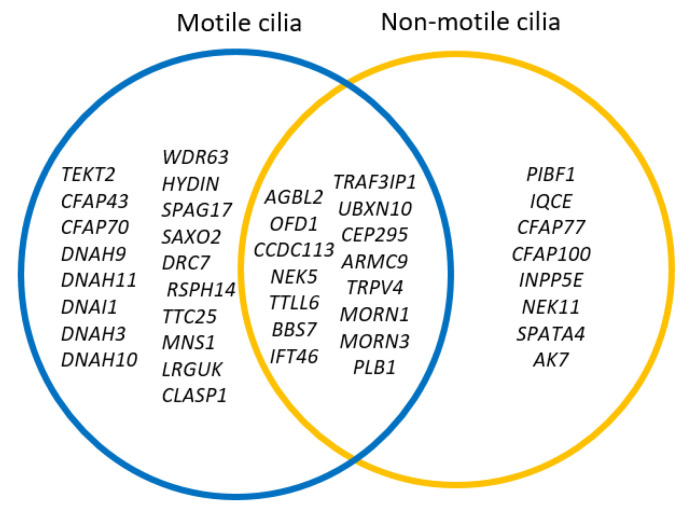
Summary of genetic anomalies found in pediatric patients with TGA. Genetic defects could be observed in the motile and non-motile cilia genes. DNAH3 and MORN3 had been associated with cilia function, although no cilia description of deletion had been reported in models.

Further, the interaction between OFD1 and PIBF1 was observed for proband T180801. Both genes have previously been implicated congenital defects in related syndromic entities OMIM Phenotype 311,200 and 617,767, respectively; thus, their implication in TGA requires further analysis. A complex interaction among DNAH9, DNAH3, WDR63, and CFAP70/TTC18 gene variants was revealed for the T180901 proband, suggesting a deleterious aggregation integration. Among them, *DNHA9* had been associated with motile cilia function and abnormal heart defects [26,27,28]; therefore, this interaction could be relevant to the pathogenesis of TGA in this patient. Regarding the T181001, a weak association between *DNAH11* and *OFD1* gene variants was found. Finally, DNAI1 and SPAG17 variants were biologically associated with the T181101 proband. DNAI1 anomalies have been previously associated with TGA [8], while the central pair associated protein SPAG17 has been associated only with Primary Ciliary Dyskinesia (PCD). Finally, the relationship between gene anomalies related to motile and non-motile cilia can be observed in Figure 3. Several genes could be related to motility or signaling in primary cilia in general; others are related to ciliogenesis, and signaling can be found in both types of cilia. This evidence is relevant and suggest that a single type of cilia cannot rule the etiopathogenesis of the transposition of the great arteries.

Finally, the effect of cilia functions on the described genes can be found in Table 5; in general, deletion models displayed several effects on cilia function or organization; despite DNAH9 defects, IFT46 and OFD1 anomalies have been associated with node cilia defects in embryo development. Other motile cilia found in this study have been associated with abnormal beating, a process that could be related to laterality and eventually to heart defects.

## 4. Discussion

The transposition of the great arteries is a complex genetic disease whose pathogenesis has not been fully elucidated. Although this disease has been associated with genetic anomalies related to the laterality establishment, its relevance in subjects with situs solitus is still a matter of discussion [2,3]. The presence of several genetic anomalies in our patients provides the context for a change in the paradigm in the genetic counseling of TGA.

Firstly, we observed gene anomalies related to Joubert and Kabuki syndromes. The presence of *KMT2D* deleterious variants suggests that epigenetic alterations could be relevant to TGA pathogenesis, as has previously been suggested for other genetic syndromes, such as MRFACD (OMIM-P 608771) [38]. To support this notion, it is necessary to analyze the presence of *KMT2D* variants in TGA patients—even those lacking the distinctive phenotype. With respect to the Joubert (Jb) syndrome-related gene, *PIBF1*, the fact that a deleterious variant c.1214G>A (p.R405Q) was found in two heterozygous patients (*p* = 6.02 × 10^−3^) suggests its relevance in TGA etiology. Although this variant is likely-pathogenic and associated with Joubert syndrome in *ClinVar*, it has been reported in a heterozygous compound context with a genomic deletion encompassing *PIBF1* coding regions [39].

Despite the consistent implication of *NODAL-GDF1-DAND5* genetic anomalies in TGA etiology [40,41], none of our patients displayed genetic defects in this developmental pathway. Since genetic clustering analyses suggest that the etiology of CHD disorders is extended beyond a single-gene alteration [9], we analyzed cilia anomalies aggregation in our probands. In general, cilia dysfunction triggers defects in the left–right animal plan patterning, leading to the development of CHD [2,42]; moreover, genes encoding cilia proteins could have non-ciliary functions that might also be significant to CHD, as has previously been revealed in the modulation of outflow tract signaling pathways [12].

Interestingly, mutations in primary cilia genes have been linked to motile dysfunction. For instance, *WDR35* heterozygous mutations, a gene involved in retrograde ciliary transport, ciliogenesis, and ciliary protein trafficking, caused motile cilia dysfunction in Sensenbrenner syndrome [43]. In this regard, we found a deleterious variant of *HYDIN*; this central apparatus protein is related to Primary Ciliary Dyskinesia (OMIM-P 608647, Ciliary Dyskinesia, Primary 5) but not to the randomization of the left-right body or CHD. Thus, the biological relevance of this variant should be interpreted in combination with the *DNAH9* missense and splicing variants found in the same proband.

On the other hand, outer dynein arm anomalies are the most common cause of PCD, a disorder related to TGA patients with abnormal LRA organ disposition [44]. DNAH9 and DNAH11 are required for the assembly and function of distal outer dynein arms; in fact, *DNAH11* mutations could result in abnormal ciliary ultrastructure and hyperkinetic ciliary beating associated with congenitally corrected TGA (ccTGA) [45], while *DNAH9* genetic anomalies are linked to either laterality defects, subtle respiratory ciliary-beating defects, and ccTGA [27,46]. Intriguingly, the situs solitus T180401 proband, which carries both a heterozygous compound *DNAH9* variant and a *HYDIN* homozygous variant, did not display any observable clinical manifestation of a ciliopathy.

We also found some anomalies in other genes related to motile ciliary function. A heterozygous nonsense variant was found in the *DNAH11*, which was linked to primary ciliary dyskinesia 7 (CILD7; OMIM-P 611884). Furthermore, a heterozygous variant was found in the CILD35-associated gene, *ODAD4/TTC25* (OMIM-P 617092). It is worth noting that defective *TTC25* has been associated with immotile nodal cilia and missing leftward flow via particle image velocimetry, lack of ODAs, and the ODA docking complex [47]. Therefore, this variant might affect cilia function somehow, and further investigation is required to determine the mechanism. The physical interaction of abnormal genetic variants suggested needs validation using assays such as a yeast two-hybrid one-on-one screening, as was previously described for *DNAH9* anomalies [27]. Parallelly, the clustering biological relevance requires further study using molecular dissection in models such as *Paramecium*, *Chlamydomonas*, or engineered retinal pigment epithelial tissue [48,49,50], which have been shown to be relevant for deciphering ciliary beating mechanisms, structures, and anchoring and ciliogenesis; and these could be valuable tools to validate candidate genes for ciliopathies. Examples of this dissection are the identification of the cilia localization of ARMC9 and CEP104 or the role of CFAP43 and CFAP70 in cilia, both using the single-celled models, and the use of techniques such as CRISPR-Cas9 to mimic the genetic variants to identify their roles in cilia function [20,28,51,52].

Furthermore, the PIBF1 aggregation in the cohort is interesting. PIBF1 had been associated with non-motile cilia assembly, and its absence has been associated with a diminished number of ciliary cells [35]. The presence of a deleterious PIBF1 variant could be suggestive of the role of cilia in TGA downstream of the early laterality establishment. In the heart, primary cilia regulate cardiogenesis via the modulation of several signaling pathways, including platelet-derived growth factor receptor-α (Pdgfra). Pdgfra and its absence has been related to this CHD [53,54].

Despite this, a central role for cilia in TGA pathogenesis was previously suggested by clinical studies where ciliary dysfunction was a predominant feature in patients [55]. Consistent with this hypothesis, a TGA exome-based analysis unveiled the enrichment of cilia-related pathways [8]. Although apparent stochastic findings and interaction were reported in half of our patients, globally we found no enrichment of any ciliary processes shared between subjects, neither in terms of motile or non-motile cilia, ciliogenesis, ciliary compartmentalization, or ciliary trafficking, that could be consistently related to TGA. Although a couple of patients displayed interactions between ciliary genes, other cilia-associated anomalies, such as airway dysfunction, were not evaluated; hence, its functional relevance requires more analysis.

Intriguingly, a recent WGS analysis showed that abnormalities in cilia-related genes were similar between TGA patients and controls [56]; in this context, the clustering of several variants in cilia genes could be relevant to identifying susceptibility genotypes that could increase the familiar risk related to cilia defects. Interestingly, a recent report described familial co-segregation of ccTGA and dextro-TGA [57]. ccTGA has been consistently associated with laterality defects [2,3], and these findings suggest a common pathogenic pathway involving laterality genes in both defects. This pathway could be related to other CHD, such as atrial/ventricular septal defects. Furthermore, a genetic anomalies integration analysis in each patient is required to determine the relevance in a personalized way as other cilia abnormalities could be relevant to diagnosis and the patient’s prognosis. As a result, in isolated and sporadic TGA, the pathogenesis could be more closely associated with an intrinsic abnormal heart development than a cilia-related abnormality.

In general, it is essential to state that it is widely accepted that variant findings require Sanger sequencing validation despite the constant improving accuracy of next-generation sequencing (NGS). Despite this, it has been suggested that high-quality reads in NGS permit a high concordance of the findings with Sanger analysis, which can be as high as 100% [58,59,60]. This concordance is essential as the translational impact of NGS variant findings could be earlier, faster, and cheaper as strict NGS quality controls standards are set.

Even though this study has limitations, the evidence presented suggests that genetic anomalies clustering is more of a risk-influencing genetic factor for non-syndromic CHD than a causative one in the transposition of the great arteries. Therefore, TGA-associated heterozygous genetic anomalies in cilia genes might cause only subtle alterations that may predispose one to the appearance of the TGA. However, these findings require confirmation and further analysis in situs solitus patients and patients with isomerism features.

## 5. Conclusions

Our study provides evidence that the clustering of anomalies in cilia genes involved in motile and non-motile cilia could underlie TGA pathogenesis, suggesting a complex and heterogeneous genetic architecture and underpinning the genetic interaction analyses as part of the genetic counseling strategy.

## Figures and Tables

**Table 1:** Abnormalities in genes previously related to TGA. Missense and splicing anomalies were found in patients with transposition of the great arteries. NODAL/GDF1-DAND5 genetic anomalies are mostly non-deleterious: while other previously cilia-related genes present features of deleteriousness.

Patients	Gene	dbSNP	NT Change	CQ	AA Change	SIFT	PP2. HumDiv	PP2. HumVar	Mutation Assessor	Allele Freq	Mend	OMIM	Del
T180101	*KDM6A*	rs780238270	c.232C>T	M	p.R78C	T (0.15)	B (0.05)	B (0.009)	L (1.31)	3.95 × 10^−4^ *	HEMY	XLD	No
	*FOXH1*	rs899189505	c.187G>A	M	p.V63I	T (0.69)	B (0.251)	B (0.083)	N (−1.435)	5.81 × 10^−5^	HE	NR	No
T180201	*GDF1*	rs944730356	c.404C>T	M	p.A135V	T (0.54)	B (0.015)	B (0.008)	N (0.6)	0.001301	HE	AD/AR	No
	*KMT2D*	New	c.547C>T	M	p.P183S	T (0.62)	PD (0.959)	PD (0.6)	N (0.55)	New	HE	AD	Yes
	*MEGF8*	rs769862975	c.1315C>T	M	p.R439W	D (0)	PD (1)	PD (0.948)	M (2.27)	6.13 × 10^−4^	HE	AR	Yes
T180301	*KMT2D*	rs201628357	c.15686G>A	M	p.R5229H	D(0.04)	PD (1)	PD (0.98)	N (0.255)	2.49 × 10^−5^ *	HE	AD	Yes
T181001	*MEGF8*	rs1281253733	c.2344C>T	M	p.R782W	D (0.085)	PD (0.962)	D (0.898)	N (0.345)	8.11 × 10^−6^	HE	AR	Yes
T181101	*DNAI1*	rs771320807	c.203G>A	M	p.R68Q	D (0.0)	PD (0.998)	PD (0.917)	M (2.455)	2.89 × 10^−5^	HE	AR	Yes
	*DISC1*	rs753171376	c.1852C>G	M	p.P618A	T (0.08)	PD (0.995)	PD (0.865)	M (2.24)	2.83 × 10^−4^ *	HE	AR	Yes
	*CCDC65*	rs200575863	c.470+3A>G	S	Splice acceptor	16.55 CAD PHRED (No deleterious)				8.52 × 10^−4^	HE	AR	No
T181201	*SLC4A1*	rs2285644	c.2561C>T	M	p.P854L	D (0.04)	PD (0.823)	B (0.140)	M (2.66)	0.04128	HE	Yes	Yes
	*CLASP1*	rs373752835	c.170C>T	M	p.S57F	T (0.08)	PD (0.985)	PD (0.55)	M (2.08)	2.89 × 10^−5^	HE	No	Yes
T181401	*PLB1*	rs745799206	c.2089-2A>G	S	Splice acceptor	33 CAD PHRED (Deleterious)				8.674 × 10^−5^	HE	NR	Yes
	*CLASP1*	New	c.1110T>G	M	p.D370E	T (0.92)	B (0)	B (0.001)	N (−0.175)	New	HE	No	No

dbSNP: Single Nucleotide Polymorphism database; AA: Amino acid; NT: Nucleotide; CQ: Consequence of genetic variant; M: Missense, N: Nonsense; F: Frameshift SIFT: PP2: Polyphen2; Freq: Frequency; Mend: Mendelian inheritance; OMIM: Online Mendelian Inheritance of Man; PD: Probably/Possibly damaging; B: Benign; L: Lo; M: Medium; H: High; N: Neutral; T: Tolerated; Del/D: Deleterious. AD: Autosomic dominant; AR: Autosomic recessive; NF: Not found; HETO: Heterozygous; HOMO: Homozygous; HEMY: Hemizygous; Allele Freq: Allele frequency in Latin population or if data is not available an asterisk (*) represent global frequency.

**Table 2:** Anomalies in motile ciliary genes in TGA patients. Missense, splicing, and nonsense anomalies of ciliary genes found in patients.

Patient	Gene	dbSNP	NT Change	CQ	AA Change	SIFT	PP2. HumDiv	PP2. HumVar	Mutation Assessor	Allele Freq	OMIM	Mend	Variant Associated
T180101 Male	*DRC7*	rs199828087	c.1819C>T	M	p.R607C	D (0.04)	D (0.91)	PD (0.99)	L (1.81)	2.83 × 10^−5^	NR	HE	No
	*TTC25*	rs782333806	c.218C>T	M	p.S73L	D (0)	PD (1)	PD (0.97)	M (2.54)	5.94 × 10^−4^	AR	HE	No
	*RSPH14*	rs780971104	c.488A>G	M	p.E163G	D (0)	B (0.02)	B (0.02)	M (2.71)	4.63 × 10^−4^	NR	HE	No
T180201 Male	*NEK5*	rs35465612	c.1420C>T	M	p.R474C	D (0)	PD (1)	PD (0.95)	M (2.44)	9.27 × 10^−3^	NR	HE	No
	*LRGUK*	rs140175129	c.2044C>T	M	p.R682C	D (0)	PD (0.96)	B (0.27)	N (0)	3.44 × 10^−2^	NR	HE	No
	*CFAP43*	rs150378110	c.3935G>A	M	p.R1312H	T (0.12)	PD (0.99)	PD (0.85)	M (2.59)	1.21 × 10^−3^	AD/R	HE	No
	*DNAH11*	rs1243678738	c.12577C>T	N	p.Q4193/Stop	48 CAD PHRED (Deleterious)				2.897 ×10^−5^	HE	AR	No
T180301 Male	*DNAH10*	rs779897384	c.1468C>A	M	p.P490T	D (0.01)	PD (0.99)	PD (0.97)	M (3.04)	1.98 × 10^−3^	NR	HE	No
	*DNAH3*	rs182462514	c.608T>C	M	p.M203T	T (0.1)	PD (0.45)	B (0.07)	M (2.17)	3.95 × 10^−3^	NR	HE	No
T180401 Male	*HYDIN*	New	c.3332C>T	M	p.P1111L	D (0)	PD (0.99)	PD (0.98)	M (2.76)	New	AR	HO	No
	*DNAH9*	rs139596704	c.3050A>G	M	p.Y1017C	D (0)	PD (0.98)	PD (0.82)	M (2.93)	5.30 × 10^−2^	AR	HE	No
	*DNAH9*	rs777167537	c.5151+1G>A	S	Splice acceptor	34 CAD PHRED (Deleterious)				1.1 × 10^−4^	AR	HE	No
T180701 Female	*WDR63*	rs1056616254	c.1742C>A	M	p.T581N	D (0)	PD (0.98)	PD (0.64)	M (2.58)	1.19 × 10^−5^ *	NR	HE	No
	*CFAP43*	rs117768807	c.589G>A	M	p.V197M	D (0)	PD (0.99)	PD (0.97)	L (1.76)	3.05 × 10^−4^	AD/R	HE	No
	*CCDC113*	rs144246110	c.300A>T	M	p.K100N	D (0)	PD (1.0)	PD (0.99)	M (2.85)	2.03 × 10^−3^	NR	HE	No
T180801 Male	*DNAH10*	rs755673190	c.8228C>T	M	p.P2743L	D (0.05)	B (0.005)	B (0.005)	M (2.53)	2.83 × 10^−5^	NR	HE	No
	*OFD1*	rs779051357	c.2482T>G	M	p.F828V	T (0.07)	PD (0.90)	PD (0.59)	M (2.43)	5.17 × 10^−5^	XLD	HEMY	No
T180901 Male	*WDR63*	rs138379333	c.922G>A	M	p.A308T	D (0.04)	PD (0.79)	B (0.14)	M (2.49)	5.39 × 10^−2^	NR	HE	No
	*CFAP70*	rs575812060	c.3079T>A	M	p.C1027S	D (0.02)	PD (0.98)	PD (0.90)	M (2.43)	2.60 × 10^−3^	AR	HE	No
	*DNAH3*	rs141197402	c.8597A>G	M	p.H2866R	D (0)	PD (0.83)	PD (0.49)	L (1.29)	1.87 × 10^−3^	NR	HE	No
	*DNAH9*	rs267604735	c.7150G>A	M	p.G2384R	D (0.04)	PD (0.99)	D (0.91)	H (3.71)	5.79 × 10^−5^	AR	HE	No
	*ARMC9*	rs386656198	c.878C>T	M	p.T293M	T (1)	PD (1.0)	D (0.98)	M (2.33)	5.24 × 10^−4^	AR	HE	No
T181001 Female	*DNAH11*	rs199789835	c.8521A>G	M	p.S2841G	D (0.02)	PD (0.95)	PD (0.79)	M (2.95)	2.23 × 10^−4^	AR	HE	No
	*OFD1*	New	c.2610G>C	M	p.Q870H	D (0.04)	PD (0.89)	PD (0.63)	M (2.12)	New	XL	HEMY	No
	*CEP295*	rs763108226	c.512C>T	M	p.P171L	D (0.04)	PD (0.72)	B (0.25)	NF	5.37 × 10^−3^	ND	HE	No
T181101 Male	*SPAG17*	rs140959339	c.430C>T	M	p.R144W	D (0)	PD (1.0)	PD (0.99)	M (2.70)	2.37 × 10^−2^	NR	HE	No
	*DNAI1*	rs771320807	c.203G>A	M	p.R68Q	D (0)	PD (0.99)	PD (0.91)	M (2.45)	2.89 × 10^−4^	AR	HE	No
	*MNS1*	rs549395315	c.605delA	F	p.L202SfsTer	29.2 CAD PHRED (Deleterious)				NF	AR	HE	No
T181201 Male	*SPAG17*	rs1028261558	c.1700C>A	M	p.P567Q	D (0.01)	PD (0.97)	PD (0.84)	M (2.62)	1.74 × 10^−4^	NR	HE	No
	*DNAH9*	rs139596704	c.3050A>G	M	p.Y1017C	D (0)	PD (0.98)	PD (0.82)	M (2.93)	5.30 × 10^−2^	AR	HE	No
T181401 Male	*TEKT2*	rs144497984	c.1114C>T	M	p.R372W	D (0.01)	PD (1.0)	PD (0.99)	M (2.86)	2.21 × 10^−2^	NR	HE	No

Allele frequency in the Latin population or if data is unavailable, an asterisk (*) represents global frequency. dbSNP: Single Nucleotide Polymorphism database; AA: Amino acid; NT: Nucleotide; CQ: Consequence of genetic variants; M: Missense, N: Nonsense; F: Frameshift; SIFT: PP2: Polyphen2; Freq: Frequency; Mend: Mendelian inheritance; OMIM: Online Mendelian Inheritance of Man; PD: Probably/Possibly damaging; B: Benign; L: Lo; M: Medium; H: High; N: Neutral; T: Tolerated; Del/D: Deleterious. AD: Autosomic dominant; AR: Autosomic recessive; NF: Not found; HETO: Heterozygous; HOMO: Homozygous; HEMY: Hemizygous; Jb: Joubert; NEDY: Neuromuscular dysplasia.

**Table 3:** Genetic anomalies in non-motile cilia genes in TGA. Missense, splicing, and nonsense anomalies of ciliary genes found in patients with transposition of the great arteries.

Patient	Gene	dbSNP	NT Change	CQ	AA Change	SIFT	PP2. HumDiv	PP2. HumVar	Mutation Assessor	Allele Freq	OMIM	Mend	Variant Associated
T180101 Male	*PIBF1*	rs17089782	c.1214G>A	M	p.R405Q	D (0)	PD (1)	PD (0.99)	M (2.56)	0.1021	AR	HE	Jb
T180201 Male	*IQCE*	rs200648086	c.1688T>C	M	p.L563S	D (0)	B (0.19)	B (0.20)	M (2.31)	5.23 × 10^−4^	AR	HE	No
T180301 Male	*TTLL6*	rs184362955	c.517C>T	M	p.R173W	D (0)	PD (1.0)	PD (1.0)	H (4.64)	8.89 × 10^−3^	NR	HE	No
T180401 Male	*CFAP100*	rs149511023	c.589G>A	M	p.A197T	T (0.04)	PD (0.99)	PD (0.85)	M (2.71)	6.85 × 10^−3^	ND	HE	No
T180701 Female	*CFAP77*	rs11243798	c.551G>A	M	p.R184H	D (0.04)	PD (1)	PD (0.99)	M (2.65)	5.03 × 10^−3^	ND	HE	No
	*CFAP100*	rs754767651	c.1292G>C	M	p.R430T	D (0)	PD (0.99)	PD (0.84)	L (1.76)	3.76 × 10^−4^	ND	HE	No
T180801 Male	*PIBF1*	rs17089782	c.1214G>A	M	p.R405Q	D (0)	PD (1.0)	PD (0.96)	M (2.56)	0.1021	AR	HO	Jb
	*IQCE*	rs375144768	c.784C>T	M	p.L262F	D (0)	PD (1.0)	PD (0.99)	M (2.59)	2.60 × 10^−4^	AR	HE	No
	*INPP5E*	rs138150684	c.1360G>A	M	p.D454N	T (0.06)	PD (1.0)	PD (0.88)	M (1.99)	1.96 × 10^−4^ *	AR	HE	No
	*OFD1*	rs779051357	c.2482T>G	M	p.F828V	T (0.07)	PD (0.90)	PD (0.59)	M (2.43)	5.17 × 10^−5^	XLD	HEMY	No
T181001 Female	*OFD1*	New	c.2610G>C	M	p.Q870H	D (0.04)	PD (0.89)	PD (0.63)	M (2.12)	New	XL	HEMY	No
	*AK7*	rs746369518	c.159_170del	F	p.(Glu53_Glu56del)	0.514 LoF-Tool PD				3 × 10^−3^	AR	HE	No
T181201 Male	*PIBF1*	rs17089782	c.1214G>A	M	p.R405Q	D (0)	PD (1.0)	PD (0.99)	M (2.56)	0.1021	AR	HO	Jb

Allele frequency in the Latin population or if data is unavailable, an asterisk (*) represents global frequency. dbSNP: Single Nucleotide Polymorphism database; AA: Amino acid; NT: Nucleotide; CQ: Consequence; M: Missense, N: Nonsense; F: Frameshift; SIFT: PP2: Polyphen2; Freq: Frequency; Mend: Mendelian inheritance; OMIM: Online Mendelian Inheritance of Man; PD: Probably/Possibly damaging; B: Benign; L: Lo; M: Medium; H: High; N: Neutral; T: Tolerated; Del/D: Deleterious. AD: Autosomic dominant; AR: Autosomic recessive; NF: Not found; HETO: Heterozygous; HOMO: Homozygous; HEMY: Hemizygous; Jb: Joubert; NEDY: Neuromuscular dysplasia.

**Table 4:** Genetic anomalies in ciliogenesis and ciliary trafficking in TGA patients. Missense, splicing, and nonsense anomalies of ciliary genes found in patients with transposition of the great arteries.

Patient	Gene	dbSNP	NT Change	CQ	AA Change	SIFT	PP2. HumDiv	PP2. HumVar	Mutation Assesor	Allele Freq	OMIM	Mend	Variant Associated
T180101 Male	*SAXO2*	rs116324279	c.1111T>C	M	p.S371P	T (0.04)	PD (0.87)	PD (0.63)	M (2.14)	5.65 × 10^−4^	ND	HE	No
T180201 Male	*PIBF1*	rs17089782	c.1214G>A	M	p.R405Q	D (0)	PD (1)	PD (0.99)	M (2.56)	0.1021	AR	HE	Jb
	*UBXN10*	rs11556959	c.794A>G	M	p.H265R	D (0.01)	PD (0.98)	PD (0.82)	M (2.32)	2.60 × 10^−4^	NR	HE	No
T180301 Male	*TTLL6*	rs184362955	c.517C>T	M	p.R173W	D (0)	PD (1.0)	PD (1.0)	H (4.64)	8.89 × 10^−3^	NR	HE	No
T180701 Female	*TRPV4*	rs187864727	c.649G>T	M	p.A217S	T (0.13)	PD (1.0)	PD (0.99)	M (2.00)	6.85 × 10^−2^	AD	HE	NEDY
	*IFT46*	rs145438119	c.454C>G	M	p.P152A	D (0.01)	PD (1.0)	PD (1.0)	M (3.25)	3.16 × 10^−3^	AR	HE	No
	*MORN3*	rs782293129	c.616G>C	M	p.A206P	D (0)	PD (1.0)	PD (1.0)	M (2.87)	2.03 × 10^−4^	ND	HE	No
	*TRAF3IP1*	rs761035757	c.838C>T	M	p.R280W	D (0.01)	PD (0.99)	PD (0.91)	L (1.79)	2.56 × 10^−4^	AR	HE	No
T180801 Male	*TRPV4*	rs187864727	c.649G>T	M	p.A217S	T (0.13)	PD (1.0)	PD (0.99)	M (2.00)	6.85 × 10^−2^	AD	HE	NEDY
	*PIBF1*	rs17089782	c.1214G>A	M	p.R405Q	D (0)	PD (1.0)	PD (0.96)	M (2.56)	0.1021	AR	HO	Jb
	*OFD1*	rs779051357	c.2482T>G	M	p.F828V	T (0.07)	PD (0.90)	PD (0.59)	M (2.43)	5.17 × 10^−5^	XLD	HEMY	No
T180901 Male	*MORN1*	rs34587196	c.757C>T	M	p.R253W	D(0)	PD (1.0)	PD (0.99)	M (2.25)	6.57 × 10^−3^	NR	HE	No
	*ARMC9*	rs386656198	c.878C>T	M	p.T293M	T (1)	PD (1.0)	D (0.98)	M (2.33)	5.24 × 10^−4^	AR	HE	No
T181001 Female	*NEK11*	rs140058289	c.127G>C	M	p.V43L	D (0.01)	PD (0.93)	PD (0.52)	M (3.41)	4.80 × 10^−2^	NR	HE	No
	*OFD1*	New	c.2610G>C	M	p.Q870H	D (0.04)	PD (0.89)	PD (0.63)	M (2.12)	New	XL	HEMY	No
T181201 Male	*AGBL2*	rs7941404	c.956G>A	M	p.R319H	T (0.09)	PD (0.99)	PD (0.91)	M (2.49)	9.9 × 10^−5^ *	NR	HE	No
	*PIBF1*	rs17089782	c.1214G>A	M	p.R405Q	D (0)	PD (1.0)	PD (0.99)	M (2.56)	0.1021	AR	HO	Jb
T181401 Male	*BBS7*	rs199891330	c.508A>G	M	p.R170G	D (0.02)	PD (1.0)	PD (0.99)	M (2.66)	9.84 × 10^−4^	AR	HE	No
	*SPATA4*	rs765034017	c.599A>C	M	p.N200T	D (0.02)	PD (0.98)	PD (0.88)	M (2.17)	8.67 × 10^−5^	ND	HE	No

Allele frequency in the Latin population or if data is unavailable, an asterisk (*) represents global frequency. dbSNP: Single Nucleotide Polymorphism database; AA: Amino acid; NT: Nucleotide; CQ: Consequence; M: Missense, N: Nonsense; F: Frameshift; SIFT: PP2: Polyphen2; Freq: Frequency; Mend: Mendelian inheritance; OMIM: Online Mendelian Inheritance of Man; PD: Probably/Possibly damaging; B: Benign; L: Lo; M: Medium; H: High; N: Neutral; T: Tolerated; Del/D: Deleterious. AD: Autosomic dominant; AR: Autosomic recessive; NF: Not found; HETO: Heterozygous; HOMO: Homozygous; HEMY: Hemizygous; Jb: Joubert; NEDY: Neuromuscular dysplasia.

**Table 5:** Global effect of genetic anomalies in cilia. Deletion of cilia genes influences the process of cilia function and ciliogenesis. ODA: Outer dynein arm, IDA: Inner dynein arm.

Cilia Gene	Region Affected	Genetic Alteration Effect	References
*CFAP43*	Axonemal	Lower beating frequency	[29]
*CFAP70/TTC18*	ODA-Central pair	Reduced beating frequency	[28]
*DNAH11*	ODA	Hyperkinetic beating	[30]
*DNAH9*	ODA	Lower beating frequency, loss of outer dynein arm structures	[27,31]
*DNAI1*	ODA	Fewer actively beating cilia, loss of outer dynein arm structures	[32]
*HYDIN*	Central pair	Cilia is unable to bend normally; reduced beat frequency	[33]
*IFT46*	IFT subcomplex B	Reduced length and number of cilia	[18]
*OFD1*	Centriole	Lack of cilia in the embryonic node	[34]
*PIBF1*	Cilia assembly	Non-motile cilia assembly, Reduced number of cilia	[35]
*SPAG17*	Central pair	Central pair structural abnormalities	[36]
*TRAF3IP1/IFT154*	IFT subcomplex B	Absence of cilia, abnormal ciliogenesis	[37]
*WDR63/DNAI3*	IDA	Disorganization of cilia	[23]

## Data Availability

The data supporting this study’s findings are available at https://www.ncbi.nlm.nih.gov/sra/PRJNA865734.

## Supplementary Materials

The following supporting information can be downloaded at: https://www.mdpi.com/article/10.3390/genes13091662/s1, Table S1: List of cilia genes and previously reported genes analyzed in the patients with transposition of the great arteries; Table S2: Complete gene abnormalities of ciliary genes in TGA; Table S3: Protein clusters of selected genes in TGA patients. Figure S1: Genetic anomalies aggregation of transposition of the great arteries by String.
