# Supplementary material for: Clustering of Genetic Anomalies of Cilia Outer Dynein Arm and Central Apparatus in Patients with Transposition of the Great Arteries
Source: Genes (Basel). 2022 Sep 16;13(9):1662. doi: 10.3390/genes13091662 (PMC9498580; doi:10.3390/genes13091662)
Supplement: Supplementary file 1 [file genes-13-01662-s001.zip › genes-1887476-supplementary.pdf]

Table S1: List of cilia genes and previously reported genes analyzed in the patients with transposition of the great arteries.

Motile cilia genes:

Central pair:
HYDIN, CCDC180, CFAP54, SPAG6, SPAG17, WDR93

Nexin-dynein regulator complex:
CCD39, CCDC65, CCDC40, DRC1, GAS8, CCDC164, CCDC40, CCDC65, DRC3, DRC7, FBXL13, TCTE1

Radial spoke:
DYDC2, DNAJB13, RSPH1, RSPH2, RSPH3, RSPH4A, RSPH9, PPIL6, RPON1L RSPH10B, RSPH10B2, RSPH14

Centrosomal:
CCDC113, C4orf47, CEP295, CCDC113, C4orf47, CEP104, CEP120, CEP162, CEP19, CEP192, CEP44, CEP76, CEP83, CEP83, CEP97
Centriole :
CFAP53, CFAP65, DEUP1, KIF24, NEK5, OFD1, POC1B
Centriolar satellite:
CCDC81, CCDC96, CCDC146, NEK10, TEX9

Ciliary tip:
ARMC9, CEP104

Outer dyneir arm:
CFAP70
Heavy chain
DNAH1, DNAH9, DNAH11, DNAH5, DNAH8
Intermediate chain
DNAI1, DNAI2
Light Chain
DNAL1, DYNLRB2, NME9
Inner dynein arm
CASC1, ZMNYD12
Heavy chain
DNAH2, DNAH3, DNAH6, DNAH10, DNAH12
Intermediate chain
WDR63, WDR78
Light intermediate chain
DNAL1

Cytoplasm region of cilia:
PIH1D3

Dynein pre-assembly:
DNAAF1, DNAAF2, DNAAF3, DNAAF4, DNAAF5, SPAG1, ZMYND10, LRCC6, C21Orf59

Axoneme:
AKAP14, CCDC173, CCDC33, CFAP43, EFHC1, EFHC2, LRGUK, PPP1R32, PACRG, RIBC2, SPACA9. TTCT29, TEKT1, WDR66
Periaxoneme
SPATA6, TEKT3
Outer dense fiber
TEKT2, TEK4H

Other gene related to Outer /inner dynein arm:
NME6, NME8, CLASP1

Cilia 96-nm repeat ruler:
CCDC39, CCDC40

Outer dynein arm attachment:
CCDC103
Docking complex:
ARMC4, CCDC114, CCDC151, MNS1, DZANK1
Dynein Motor Complex:
TTC25

Non-motile cilia:

Axoneme and signaling :
ADCY6, ANKS3, ANKS6, CEP41, CNG11, CNGA41, CNGB1, C2orf71, C2orf2, DCDC2, EFHC1, FLCN1, GPR161, ICK, INPP5E, IQCB1, LCA5, NEK1, OCRL, PIK3R4, PKD1, PKD2, PKD1L1, RAB23, SDCCAG8, SMO, USP9X

Dynein assembly docking :
ARMC4, CCDC114, DNAAF1, HEATR2, LRCC6, MNS1, ZMNYD10

Primary Cilia assembly:
PIBF1

Cilium tip:
CEP104, GLI2. GLI3, KIF7, SUFU

INV compartment:
EVC2, EVC, INVS, NPHP3, NEK8, IQCE

Other: Cytosol, Golgi, nucleus, etc
AK7, DDX59, GALNT11, GLIS2, PIK3R4, POMGNT1, TBC1D32, XPNPEP3, ZNF423

Transcription factor:
CRX, ZIC2

Other non motile-cilia related genes:
ALS2CR12, CFAP61, CFAP77, CFAP100, CFAP299, C9orf116, FANK1, IQCD, IQUB, LRRC34, EFCAB1, MYCBPAP, SPA17
Cilia tip
C1orf87
Golgi cilia network
PIFO
Cytoskeleton
CATIP, DCDC1, TTC23L

Transcription factors:
FOXJ1, RFX

Ciliogenesis:

Centriole or basal body:
ALMS1, ARL2BP, CENPF, CEP19, CEP41, EP78, CEP104, CEP120, CSPP1, C2CD3, C21orF2, FAM16A, FLCN, HYLS1, INTU, KIAA0556, KIZ, NEK2, NME7, OCRL1, OFD1, PIBF1, PLK4, POC1B, POC1A, RAB2B, RP2, TALPID3, TAPT1, TOPORS, TTL5, TUB, WDPCP

Cilia assembly:
ARMC2, C9orf24, CCDC151, CFAP126, CFAP300, DAW1, DNAAF1, DNAAF3, LRRC6, MAK, MARCH10, PIH1D2, PIHD3, SAXO2, UBXN10, ZYMND10

Transition zone
AHI1, B9D1, B9D2, CC2D2A. CEP900, C5orf42, IQCB1, LCA5, MKS1, NPHP1, NPHP4, RP1, RP1L1, RPGRIP, RPGRIP1. RPGRIP1L, SDCCAG8, SPATA7, TCTN1, TTN1, TCTN3, TMEM17. TMEM67.,TMEM107, TMEM138, TMEM216, TMEM231, TMEM237, TMEM67, MKS1, CCT2

Centriolar satellites
BBS4, CCDC28B, CFAP53, CEP290, KIAA0753, OFD1

Cilia base:
ARMC3, C5orf49. CCDC170, TSGA10
Basal body:
AGBL2, BBOF1, C11orf97, CABCOCO1, CCDC13, CFAF157, ENKUR, MAPK15, MORN1, PPP1R42, ARMC9

Tubulin modifying enzime:
TUB1A, TTLL6

Distal appendage:
CEP83, CEP164, SCLT1, TTBK2

Unclear or various localization:
ATXN10, CFAP52, NEK9, TULP1

Subidstal appendage:
CC2D2A

Cilia trafficking:

IFT dynein subunits:
DYNC2H1, DYNC2LI1, TCTEX1D2, WDR34, WDR35, WDR60

IFT subcomplex A:
IFT43, IFT140, WDR19, IFT122, TTC21B. IFT121, IFT139, IFT14

BBS proteins
BBSome:
ARL, BBS1, BBS2, BBS4, BBS5, BBS7, BBS8, BBS9, TTC8
Bbsome regulatoris:
BIBP1, BBS10, BBS12, CEP290, C8orf37, LZFTL1, MKKS, MKS, SDCCAG8, TRIM32,

IFT subcomplex B:
CLUAP1, ITF23, ITF57, IFT52, IFT80, IFT81, IFT172. TRAF3IP1, IFT20, IFT46, IFT52, IFT57, IFT80, IFT88. IFT172, DISC1, RABL5, TRAF3LP1. TTC26, TTC30B, KIF3A, KIF3B, KIFAP3

Cytoplasm related:
IQCG, LRRRC23, MORN3, STK33
Nuclear related:
CCNA1, CFAP45, FOXJ1, MAP3K19, NEK11, SPATA4, ZEB2

IFT Cilia regulators:
FAM58A, ICK, MAK

LIFT:
ARL13B, ARL3, PDE6D, RP2, UNC119

Ciliary receptor :
TRPV4

TGA previously related genes:

Laterality:
ZIC3, PITX2, LEFTY2, FOXH1, GDF1, ACVR2B, NODAL, CFC1, LEFTY1 CCT2, CEP57, SLC4A1, AK7, ARL13B, POC1B, DISC1, DNAI1. CLASP1. DNAAF2, MLF1, IFT43, PRKAR1A, GPR161, NME8, B9D1, TMEM260, PLB1, SLC4A1.

Heart development:
CITED2, HIF1A, TBX1, HSPG2, MED13L, ISL1, MYH6, MYH10, MEF2C

Table S2: Complete abnormalities of ciliary genes in TGA. Missense anomalies of ciliary genes found in patients with transposition of great arteries.

Patient	Gene	dbSNP	NT change	AA change	SIFT	PP2. HumDiv	PP2. HumVar	Mutation Assesor	Allele Freq	OMIM	Mend	¿Del?	Entity Associated
**T1801 Male**	*DNAH6*	rs774899113	c.637A>G	p.I213V	T (1)	B (0.01)	B (0.008)	L (1.71)	2.75E x 10^{-4}	NR	HETO	No	No
	FOXH1	rs899189505	c.187G>A	p.V63I	T (0.69)	B (0.251)	B (0.083)	N (-1.43)	5.81E x 10^{-5}	NR	HETO	No	No
	DNAH10	rs752103532	c.2489G>A	p.R830Q	T (0.37)	B (0.135)	B (0.011)	M (2.11)	1.77 x 10^{-5}	NR	HETO	No	No
	SAXO2	rs116324279	c.1111T>C	p.S371P	T (0.04)	PD (0.879)	PD (0.635)	M (2.14)	5.65 x 10^{-4}	ND	HETO	Yes	No
	WDR93	rs28727970	c.238G>A	p.A80T	T (0.37)	B (0.028)	B (0.014)	N (0.74)	3.17 x 10^{-3}	ND	HETO	No	No
	DRC7	rs199828087	c.1819C>T	p.R607C	D (0.04)	D (0.912)	PD (0.992)	L (1.81)	2.83 x 10^{-5}	NR	HETO	Yes	No
	TTC25	rs782333806	c.218C>T	p.S73L	D (0)	PD (1)	PD (0.976)	M (2.54)	5.94 x 10^{-4}	AR	HETO	Yes	No
	RSPH14	rs780971104	c.488A>G	p.E163G	D (0)	B (0.026)	B (0.028)	M (2.71)	4.63 x 10^{-4}	NR	HETO	Yes	No
	RIOK1	rs55698032	c.341G>A	p.R115Q	T (0.22)	B (0.007)	B (0.004)	L (1.2)	5.85 x 10^{-5}	NR	HETO	No	No
	PIBF1	rs17089782	c.1214G>A	p.R405Q	D (0)	PD (1)	PD (0.996)	M (2.56)	0.1021	AR	HETO	Yes	Joubert
	KATNB1	rs141820607	c.1841A>G	p.H614R	T (0.07)	B (0.42)	B (0.018)	L (0.9)	1.31 x 10^{-4}	AR	HETO	No	No
	KDM6A	rs780238270	c.232C>T	p.R78C	T (0.15)	B (0.05)	b (0.009)	L (1.31)	3.95 x 10^{-5}*	XL	HEMY	No	No
**T180201 Male**	*UBXN10*	rs11556959	c.794A>G	p.H265R	D (0.01)	PD (0.982)	PD (0.824)	M (2.32)	2.60 x 10^{-4}	NR	HETO	Yes	No
	PLB1	rs62131028	c.2710A>T	p.N904Y	T (0.13)	PD (0.966)	PD (0.942)	M (2.05)	5.96 x 10^{-3}	NR	HETO	Yes	No
	NEK5	rs35465612	c.1420C>T	p.R474C	D (0)	PD (1)	PD (0.959)	M (2.44)	9.27 x 10^{-3}	NR	HETO	Yes	No
	ALDH6A1	rs183066442	c.1604G>A	p.R535H	D LC (0)	B (0)	B (0.001)	L (1.04)	7.9 x 10^{-6} *	AR	HETO	No	No
	IQCE	rs200648086	c.1688T>C	p.L563S	D (0)	B (0.193)	B (0.207)	M (2.31)	5.23 x 10^{-4}	AR	HETO	Yes	No
	KMT2D	New	c.547C>T	p.P183S	T (0.62)	PD (0.959)	PD (0.6)	N (0.55)	New	AD	HETO	Yes	No
	MEGF8	rs769862975	c.1315C>T	p.R439W	D (0)	PD (1)	PD (0.948)	M (2.27)	6.13 x 10^{-4}*	AR	HETO	Yes	No

**T180301 Male**	*RSPH10B2*	rs2711191	c.212G>C	p.S71T	T (1)	B (0)	B (0.0)	N (-2.04)	9.51 x 10^{-4}	ND	HOMO	No	No
	RSPH10B2	rs201943687	c.1835A>G	p.Y612C	T (0.13)	PD (0.761)	B (0.24)	L (1.59)	4.24 x 10^{-3}	ND	HETO	No	No
	LRGUK	rs140175129	c.2044C>T	p.R682C	D (0)	PD (0.965)	B (0.270)	N (0)	3.44 x 10^{-2}	NR	HETO	Yes	No
	CFAP43	rs150378110	c.3935G>A	p.R1312H	T (0.12)	PD (0.997)	PD (0.855)	M (2.59)	1.21 x 10^{-3}	AD/AR	HETO	Yes	No
	DNAH10	rs779897384	c.1468C>A	p.P490T	D (0.01)	PD (0.999)	PD (0.97)	M (3.04)	1.98 x 10^{-3}	NR	HETO	Yes	No
	DNAH3	rs766114089	c.6556G>A	p.V2186M	T (0.7)	PD (0.924)	PD (0.967)	L (1.55)	4.35 x 10^{-3}	NR	HETO	No	No
	DNAH3	rs182462514	c.608T>C	p.M203T	T (0.1)	PD (0.454)	B (0.073)	M (2.17)	3.95 x 10^{-3}	NR	HETO	Yes	No
	TTLL6	rs184362955	c.517C>T	p.R173W	D (0)	PD (1)	PD (1.0)	H (4.64)	8.89X 10^{-3}	NR	HETO	Yes	No
	DZANK1	rs139700575	c.1142G>A	p.R381Q	T (0.1)	B (0.106)	B (0.018)	L (1.06)	3.59 x 10^{-2}	ND	HETO	No	No
	DZANK1	rs117740377	c.1019A>C	p.Y340S	T (0.13)	B (0.001)	B (0.0)	N-(2.82)	3.50 x 10^{-2}	ND	HETO	No	No
	STX19	rs145124418	c.70T>G	p.S24A	T (0.81)	B (0)	B (0.0)	N (0)	1.03 x 10^{-3}	ND	HETO	No	No
	IQCE	rs180961841	c.1148G>T	p.R383L	T (0.63)	PD (0.924)	B (0.366)	L (1.445)	1.074 x 10^{-3}	AR	HETO	No	No
	CEP76	rs146808596	c.985G>A	p.A329T	T (0.08)	B (0.222)	B (0.117)	L (1.54)	2.68 x 10^{-4}*	ND	HETO	No	No
	KMT2D	rs201628357	c.15686G>A	p.R5229H	D(0.04)	PD (1)	PD (0.98)	N (0.255)	2.49 x 10^{-5}**	AD	HETO	Yes	No
**T180401 Male**	*WDR35*	rs1301451424	c.721C>G	p.H241N	T (0.6)	B (0)	B (0)	L (1.08)	5.84 x 10^{-5}	AR	HETO	No	No
	CFAP100	rs149511023	c.589G>A	p.A197T	T (0.04)	PD (0.998)	PD (0.850)	M (2.715)	6.85 x 10^{-3}	ND	HETO	Yes	No
	CFAP77	rs11243798	c.551G>A	p.R184H	D (0.04)	PD (1)	PD (0.994)	M (2.65)	5.03 x 10^{-3}	ND	HETO	Yes	No
	TTC8	rs928165640	c.595A>G	p. I199V	T LC (0.26)	B (0.146)	B (0.116)	N (0.6)	4.76 x 10^{-5}	AR	HETO	No	No
	DNAH3	rs144717900	c.4878T>G	p.S1626R	D (0.01)	B (0.187)	B (0.103)	L (1.64)	6.18 x 10^{-2}	NR	HETO	No	No
	HYDIN	New	c.3332C>T	p.P1111L	D (0)	PD (0.999)	PD (0.988)	M (2.76)	New	AR	HOMO	Yes	No

	DNAH9	rs139596704	c.3050A>G	p.Y1017C	D (0)	PD (0.981)	PD (0.827)	M (2.93)	5.30 x 10^{-2}	AR	HETO	Yes	No
	MYCBPAP	rs73334009	c.886C>T	p.R296C	T (0.17)	B (0)	PD (0.556)	N (0.69)	9.96 x 10^{-4}	NR	HETO	No	No
	PRSS54	rs143446844	c.626C>T	p. T209M	T (0.27)	B (0.017)	B (0.010)	N (0.755)	5.8 x 10^{-3}	NR	HETO	No	No
**T180701 Female**	*TRPV4*	rs187864727	c.649G>T	p.A217S	T (0.13)	PD (1)	PD (0.99)	M (2.00)	6.85 x 10^{-2}	AD	HETO	Yes	Neuromusc dyspl
	WDR63	rs1056616254	c.1742C>A	p.T581N	D (0)	PD (0.984)	PD (0.649)	M (2.58)	1.19 x 10^{-5}*	NR	HETO	Yes	No
	CFAP100	rs754767651	c.1292G>C	p.R430T	D (0)	PD (0.997)	PD (0.84)	L (1.76)	3.76 x 10^{-4}	ND	HETO	Yes	No
	DNAH8	rs752099198	c.7906A>G	p.T2636A	T (0.31)	B (0.099)	B (0.101)	L (1.35)	3.73 x 10^{-4}	AR	HETO	No	No
	CFAP43	rs117768807	c.589G>A	p.V197M	D (0)	PD (0.999)	PD (0.973)	L (1.76)	3.05 x 10^{-3}	AD/AR	HETO	Yes	No
	IFT46	rs145438119	c.454C>G	p.P152A	D (0.01)	PD (1)	PD (1)	M (3.25)	3.16 x 10^{-3}	AR	HETO	Yes	No
	MORN3	rs782293129	c.616G>C	p.A206P	D (0)	PD (1)	PD (1)	M (2.87)	2.03 x 10^{-4}	ND	HETO	Yes	No
	TMEM260	rs753356578	c.469A>T	p.M157L	T (0.44)	B (0.002)	B (0.002)	L (1.01)	2.04 x 10^{-4}	AR	HETO	No	No
	BBOF1	rs190718961	c.248G>A	p.S83N	T (0.2)	B (0.001)	B (0.003)	N (0.74)	4.35 x 10^{-3}	NR	HETO	No	No
	CCDC113	rs144246110	c.300A>T	p.K100N	D (0)	PD (1)	PD (0.997)	M (2.855)	2.03 x 10^{-3}	NR	HETO	Yes	No
	DZANK1	rs139700575	c.1142G>A	p.R381Q	T (0.1)	B (0.106)	B (0.018)	L (1.06)	3.59 x 10^{-2}	ND	HETO	No	No
	DZANK1	rs117740377	c.1019A>C	p.Y340S	T (1)	B (0.01)	B (0.018)	N (-2.82)	3.50 x 10^{-2}	ND	HETO	No	No
	TRAF3IP1	rs761035757	c.838C>T	p.R280W	D (0.01)	PD (0.999)	PD (0.913)	L (1.79)	2.56 x 10^{-4}	AR	HETO	Yes	No
	MIB1	rs759658918	c.1694C>G	p.T565S	T (0.06)	PD (0.843)	PD (0.893)	L (0.9)	9.14 x 10^{-5}	AD	HETO	No	No
**T180801 Male**	*TRPV4*	rs187864727	c.649G>T	p.A217S	T (0.13)	PD (1)	PD (0.99)	M (2.00)	6.85 x 10^{-2}	AD	HETO	Yes	Neuromusc dyspl
	RSPH10B2	rs2711191	c.212G>C	p.S71T	T (1)	B (0)	B (0)	N (-2.04)	9.51 x 10^{-4}	ND	HOMO	No	No
	RSPH10B2	rs759234050	c.2510C>T	p.P837L	T (0.06)	PD (0.998)	PD (0.871)	L (1.79)	5.32 x 10^{-5}**	ND	HOMO	No	No

	DNAH10	rs755673190	c.8228C>T	p.P2743L	D (0.05)	B (0.005)	B (0.005)	M (2.53)	2.83×10^{-5}	NR	HETO	Yes	No
	DNAH10	rs199619464	c.10258G>A	p.V3420I	T (0.51)	B (0.132)	B (0.078)	N (0.11)	9.08×10^{-4}	NR	HETO	No	No
	PIBF1	rs17089782	c.1214G>A	p.R405Q	D (0)	PD (1)	PD (0.966)	M (2.56)	0.1021	AR	HOMO	Yes	Joubert
	IQCE	rs375144768	c.784C>T	p.L262F	D (0)	PD (1)	PD (0.998)	M (2.595)	2.60×10^{-4}	AR	HETO	Yes	No
	INPP5E	rs138150684	c.1360G>A	p.D454N	T (0.06)	PD (1)	PD (0.885)	M (1.995)	1.96×10^{-4}*	AR	HETO	Yes	No
	OFD1	rs779051357	c.2482T>G	p.F828V	T (0.07)	PD (0.903)	PD (0.591)	M (2.43)	5.17×10^{-5}	XLD	HEMY	Yes	No
**T180901 Male**	*MORN1*	rs34587196	c.757C>T	p.R253W	D(0)	PD (1)	PD (0.999)	M (2.25)	6.57×10^{-3}	NR	HETO	Yes	No
	WDR63	rs138379333	c.922G>A	p.A308T	D (0.04)	PD (0.792)	B (0.14)	M (2.49)	5.39×10^{-2}	NR	HETO	Yes	No
	CFAP65	rs144604393	c.3079C>T	p.R1027C	T (0.14)	B (0.481)	B (0.032)	M (2.04)	5.65×10^{-5}	AR	HETO	No	No
	CFAP100	rs199685364	c.209G>A	p.R70Q	D (0.04)	B (0.105)	B (0.027)	L (1.53)	2.82×10^{-4}	ND	HETO	No	No
	NEK11	rs141777290	c.1915C>A	p.Q639K	T (0.14)	B (0.068)	B (0.014)	M (1.995)	1.98×10^{-4}	NR	HETO	No	No
	CCDC96	rs181515863	c.721C>A	p.R241S	D (0.04)	B (0.322)	B (0.034)	L (0.805)	2.18×10^{-2}	ND	HETO	No	No
	CFAP70	rs575812060	c.3079T>A	p.C1027S	D (0.02)	PD (0.986)	PD (0.906)	M (2.43)	2.60×10^{-3}	AR	HETO	Yes	No
	ALDH6A1	rs183066442	c.1604G>A	p.R535H	D LC (0)	B (0)	B (0.001)	L (1.04)	1.75×10^{-2}	AR	HETO	No	No
	DNAH3	rs144845767	c.9329A>G	p.N3110S	T (0.07)	B (0.004)	B (0.024)	L (1.89)	1.86×10^{-3}	NR	HETO	No	No
	DNAH3	rs141197402	c.8597A>G	p.H2866R	D (0)	PD (0.833)	PD (0.499)	L (1.29)	1.87×10^{-3}	NR	HETO	Yes	No
	PRSS54	rs143678998	c.1121G>A	p.R374K	T (0.15)	PD (0.919)	B (0.214)	M (2.00)	5.08×10^{-4}	ND	HETO	Yes	No
	DNAH9	rs267604735	c.7150G>A	p.G2384R	D (0.04)	PD (0.999)	D (0.918)	H (3.71)	5.79×10^{-5}	AR	HETO	Yes	No
	MARCH10	rs141504034	c.1961G>C	p.G654A	T (0.47)	PD (0.887)	B (0.251)	L (1.84)	7.90×10^{-4}	NR	HETO	No	No
**T181001 Female**	*NPHP4*	rs202179978	c.1331G>A	p.R444Q	T (1)	B (0.008)	B (0.001)	N (-0.34)	6.79×10^{-4}	AR	HETO	No	No

	ARMC9	rs386656198	c.878C>T	p.T293M	T (1)	PD (1)	D (0.984)	M (2.33)	5.24 x 10^{-4}	AR	HETO	Yes	No
	NEK11	rs140058289	c.127G>C	p.V43L	D (0.01)	PD (0.934)	PD (0.528)	M (3.41)	4.80 x 10^{-2}	NR	HETO	Yes	No
	DNAH11	rs199789835	c.8521A>G	p.S2841G	D (0.02)	PD (0.957)	PD (0.796)	M (2.95)	2.23 x 10^{-4}	AR	HETO	Yes	No
	FBXL13	New	c.253C>A	p.H85N	T (0.2)	B (0.204)	B (0.068)	N (0.69)	New	NR	HETO	No	No
	ENKUR	rs144669094	c.372T>G	p.I124M	B (0.06)	B (0.204)	B (0.055)	M (2.32)	7.96E-6*	NR	HETO	No	No
	DNAH3	rs766114089	c.6556G>A	p.V2186M	T (0.7)	PD (0.924)	B (0.342)	L (1.55)	4.35 x 10^{-3}	NR	HETO	No	No
	DRC3	rs761307066	c.652C>G	p.L218V	T (0.57)	B (0.001)	B (0.003)	N (0.67)	1.40 x 10^{-3}	NR	HETO	No	No
	NRK	rs192589373	c.2365C>T	p.P789S	T (0.25)	B (0)	B (0)	N (0)	4.39 x 10^{-2}	NR	HETO	No	No
	TTLL6	rs149873082	c.893G>A	p.R298H	T (0.93)	B (0)	B (0)	N (0.205)	2.23 x 10^{-3}	NR	HETO	No	No
	OFD1	New	c.2610G>C	p.Q870H	D (0.04)	PD (0.899)	PD (0.639)	M (2.125)	New	XL	HEMY	Yes	No
	KIF3B	rs1166002310	c.1666C>T	p.H556Y	D (0.03)	B (0.331)	B (0.112)	L (0.895)	1.40 x 10^{-4}	AD	HETO	No	No
	CEP295	rs763108226	c.512C>T	p.P171L	D (0.04)	PD (0.728)	B (0.25)	NF	5.37 x 10^{-3}	ND	HETO	Yes	No
	MEGF8	rs1281253733	c.2344C>T	p.R782W	D (0.085)	PD (0.962)	D (0.898)	N (0.345)	8.11 x 10^{-6}	AR	HETO	Yes	No
**T181101 Male**	*SPAG17*	rs140959339	c.430C>T	p.R144W	D (0)	PD (1)	PD (0.99)	M (2.70)	2.37 x 10^{-2}	NR	HETO	Yes	No
	RIOK1	rs1228728066	c.333G>C	p.K111N	T (0.12)	B (0.129)	B (0.067)	M (1.985)	5.78 x 10^{-5}	NR	HETO	No	No
	DNAI1	rs771320807	c.203G>A	p.R68Q	D (0)	PD (0.998)	PD (0.917)	M (2.45)	2.89 x 10^{-5}	AR	HETO	Yes	No
	DYNC2H1	rs200264343	c.12937A>G	p.N4313D	D (0.09)	B (0.064)	B (0.015)	N (0.34)	1.19 x 10^{-3}	AR/DR	HETO	No	No
	CCDC33	rs189088694	c.52G>A	p.A18T	T (1)	B (0.01)	B (0.01)	N (-1.245)	2.21 x 10^{-3}	NR	HETO	No	No
	C2CD3	rs150676400	c.6343C>T	p.P2115S	T (0.51)	B (0.01)	B (0.01)	N (0.77)	5.00 x 10^{-3}	AR	HETO	No	No
**T181201 Male**	*SPAG17*	rs1028261558	c.1700C>A	p.P567Q	D (0.01)	PD (0.979)	PD (0.848)	M (2.62)	1.74 x 10^{-4}	NR	HETO	Yes	No

	PLB1	rs62131028	c.2710A>T	p.N904Y	T (0.13)	PD (0.996)	PD (0.942)	M (2.05)	5.96×10^{-3}	NR	HOMO	Yes	No
	RSPH10B2	rs2711191	c.212G>C	p.S71T	T (1)	B (0)	B (0.0)	N (-2.04)	9.51×10^{-4}	ND	HOMO	No	No
	AGBL2	rs7941404	c.956G>A	p.R319H	T (0.09)	PD (0.999)	PD (0.917)	M (2.49)	9.9E-5*	NR	HETO	Yes	No
	CFAP54	rs1423580555	c.1964A>G	p.H655R	T (0.07)	B (0.124)	B (0.124)	NF	9.37E-6*	ND	HETO	No	No
	DNAH9	rs139596704	c.3050A>G	p.Y1017C	D (0)	PD (0.981)	PD (0.827)	M (2.93)	5.30×10^{-2}	AR	HETO	Yes	No
	PIBF1	rs17089782	c.1214G>A	p.R405Q	D (0)	PD (1)	PD (0.996)	M (2.56)	0.1021	AR	HOMO	Yes	Joubert
	LZTFL1	rs751968807	c.724A>G	p.T242A	T (0.47)	B (0.01)	B (0.02)	N (0.4)	0.001721	AR	HETO	No	No
	RPGRIP1L	New	c.2957A>G	p.D986G	T (0.035)	B (0)	B (0.0)	N (0)	New	AR	HETO	No	No
	CEP192	rs117901677	c.6737T>C	p.F2246S	T (0.24)	B (0.036)	B (0.030)	NF	5.56×10^{-3}	NR	HETO	No	No
**T181401 Male**	*TEKT2*	rs144497984	c.1114C>T	p.R372W	D (0.01)	PD (1)	PD (0.99)	M (2.86)	2.21×10^{-2}	NR	HETO	Yes	No
	CCDC13	rs34234072	c.164A>G	p.D55G	D (0.05)	B (0.001)	B (0.003)	L (1.64)	4.74×10^{-3}	ND	HETO	No	No
	BBS7	rs199891330	c.508A>G	p.R170G	D (0.02)	PD (1)	PD (0.994)	M (2.66)	9.84×10^{-4}	AR	HETO	Yes	No
	SPATA4	rs765034017	c.599A>C	p.N200T	D (0.02)	PD (0.984)	PD (0.884)	M (2.17)	8.67×10^{-5}	ND	HETO	Yes	No
	IFT140	New	c.60T>G	p.F20L	T (0.52)	B (0)	B (0.0)	N (-0.97)	New	AR	HETO	No	No
	DZANK1	rs79400176	c.848G>A	p.S283N	T (0.47)	B (0.077)	B (0.026)	L (1.76)	3.76×10^{-3}	ND	HETO	No	No
	TTBK2	rs200773248	c.1936C>G	p.Q646E	T (0.05)	B (0.304)	B (0.072)	M (2.38)	3.92×10^{-4}	AD	HETO	No	No
	CEP44	rs139331696	c.556A>G	p.I186V	T (0.61)	B (0.017)	B (0.11)	L (1.245)	6.75×10^{-4}	ND	HETO	No	No
	EVC2	rs754380041	c.209G>T	p.G70V	T (0.19)	B (0.082)	B (0.276)	N (0)	4.57×10^{-4}	AR/AD	HETO	No	No
	KIFAP3	rs1203092409	c.646C>G	p.H216D	T (0.07)	B (0.44)	B(0.138)	M (2.05)	3.25×10^{-5}	NR	HETO	No	No

The algorithms could display several differences for a single variant, and the deleteriousness should be interpreted in this context. *TRPV4* and *PIBF1* genetic variants had been previously reported to other genetic diseases. dbSNP: Single Nucleotide Polymorphism database ; AA: Amino acid; NT: Nucleotide; SIFT: PP2: Polyphen2; Freq: Frequency; Mend: Mendelian inheritance; OMIM: Online Mendelian Inheritance of Man; PD: Probably/Possibly damaging; B: Benign; L: Lo; M: Medium; H: High; N: Neutral; T: Tolerated; Del/D: Deleterious. AD: Autosomic dominant; AR:

Autosomic recessive; NF: Not found; HETO: Heterozygous; HOMO: Homozygous; HEMY: Hemizygous; Neuromusc dyspl: Neuromuscular dysplasia. Allele frequency in latin population or if data is not available an asterisk (*) represent global frequency

Table S3: Protein clusters of selected genes in TGA patients. The analysis was performed in String using the Markov clustering algorithm.

#Clustering method	Cluster number	Cluster color	Gene count	Protein name	protein identifier
MCL	1	Red	8	DNAH10	9606.ENSP00000386770
MCL	1	Red	8	DNAH11	9606.ENSP00000475939
MCL	1	Red	8	DNAH3	9606.ENSP00000261383
MCL	1	Red	8	DNAH9	9606.ENSP00000262442
MCL	1	Red	8	DNAI1	9606.ENSP00000480538
MCL	1	Red	8	TTC18	9606.ENSP00000310829
MCL	1	Red	8	TTC25	9606.ENSP00000478589
MCL	1	Red	8	WDR63	9606.ENSP00000294664
MCL	2	Brown	7	CCDC135	9606.ENSP00000353942
MCL	2	Brown	7	CCDC65	9606.ENSP00000312706
MCL	2	Brown	7	HYDIN	9606.ENSP00000377197
MCL	2	Brown	7	MNS1	9606.ENSP00000260453
MCL	2	Brown	7	NEK5	9606.ENSP00000347767
MCL	2	Brown	7	TEKT2	9606.ENSP00000207457
MCL	2	Brown	7	TTLL6	9606.ENSP00000377043
MCL	3	Olive	5	BBS7	9606.ENSP00000264499

MCL	3	Olive	5	DISC1	9606.ENSP00000355593
MCL	3	Olive	5	IFT46	9606.ENSP00000264020
MCL	3	Olive	5	TRAF3IP1	9606.ENSP00000362424
MCL	3	Olive	5	UBXN10	9606.ENSP00000364240
MCL	4	Green	3	CCDC113	9606.ENSP00000219299
MCL	4	Green	3	SPAG17	9606.ENSP00000337804
MCL	4	Green	3	SPATA4	9606.ENSP00000280191
MCL	5	Blue	2	OFD1	9606.ENSP00000344314
MCL	5	Blue	2	PIBF1	9606.ENSP00000317144
MCL	6	Light Sky Blue	2	AK7	9606.ENSP00000267584
MCL	6	Light Sky Blue	2	CFAP43	9606.ENSP00000349568
MCL	7	Medium Blue	2	CCDC37	9606.ENSP00000344749
MCL	7	Medium Blue	2	MORN3	9606.ENSP00000347486

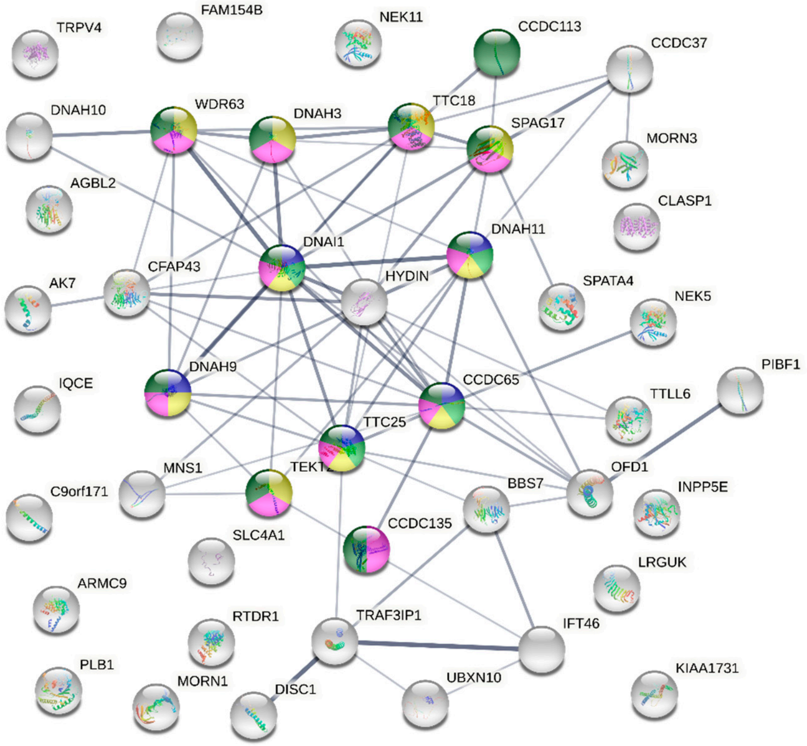

Local network cluster (STRING)

cluster	description	count in network	strength	false discovery rate
CL:10812	Axonemal dynein complex	3 of 15	1.95	0.0048
CL:10696	Kartagener syndrome	3 of 16	1.92	0.0050
CL:10690	Primary ciliary dyskinesia, and axonemal central apparatus	7 of 41	1.88	1.08e-08
CL:10692	Primary ciliary dyskinesia	5 of 30	1.87	1.05e-05
CL:10694	Primary ciliary dyskinesia	4 of 24	1.87	0.00028
CL:10689	Primary ciliary dyskinesia, and COPI-independent Golgi-to-E...	10 of 78	1.76	6.49e-12
CL:10687	Mixed, incl. primary ciliary dyskinesia, and copi-independent...	11 of 107	1.66	3.43e-12
CL:10686	Mixed, incl. primary ciliary dyskinesia, and copi-independent...	12 of 130	1.61	1.02e-12

Figure S1: Genetic anomalies aggregation of transposition of the great arteries by String. Further Ciliary dyskinesia, genetic variants are accumulated in COPI independent Golgi to ER retrograde traffic.
